# Women Doctors and the Ban on Marriage

**Published:** 1914-04-11

**Authors:** E. Honor Bone


*To the Editor of* The Hospital.

### Women Doctors and the Ban on Marriage.

*To the Editor of* The Hospital.

Sir,—With reference to the public-health work of married medical women I consider that each case requires to be settled on its own merits, and that it is the woman herself who should be the judge.

This does not leave the employer without legitimate rights, as he is perfectly entitled to dismiss the woman either before or after marriage if he has reason to be dissatisfied with her work.—Yours faithfully,

E. Honor Bone, M.D.

